# Training Reduces Stress in Human-Socialised Wolves to the Same Degree as in Dogs

**DOI:** 10.1371/journal.pone.0162389

**Published:** 2016-09-09

**Authors:** Angélica da Silva Vasconcellos, Zsófia Virányi, Friederike Range, César Ades, Jördis Kristin Scheidegger, Erich Möstl, Kurt Kotrschal

**Affiliations:** 1 Program of Post-Graduation in Vertebrate Biology, Pontifical Catholic University of Minas Gerais, Belo Horizonte, Minas Gerais, Brazil; 2 Institute of Psychology, University of São Paulo, São Paulo, São Paulo, Brazil; 3 Wolf Science Center, Enrstbrunn, Austria; 4 Messerli Research Institute—University of Veterinary Medicine Vienna, Medical University of Vienna, University of Vienna, Vienna, Austria; 5 Department of Biomedical Sciences, Unit of Physiology, Pathophysiology and Experimental Endocrinology, University of Veterinary Medicine, Vienna, Austria; 6 Department of Behavioural Biology, University of Vienna, Vienna, Austria; Faculty of Animal Sciences and Food Engineering, University of São Paulo, BRAZIL

## Abstract

The welfare of animals in captivity is of considerable societal concern. A major source of stress, especially for wild animals, is the lack of control over their environment, which includes not being able to avoid contact with human beings. Paradoxically, some studies have shown that interactions with human beings may improve the welfare of wild animals in captivity. Here, we investigated the behavioural (behaviours indicative of cooperation or stress) and physiological (variations in salivary cortisol concentrations) effects of the increasingly used practice of training wild animals as a way to facilitate handling and/or as behavioural enrichment. We evaluated the effects of indoor training sessions with familiar caretakers on nine human-socialised individuals of a wild species, the wolf (*Canis lupus*), in comparison to nine individuals of its domesticated form, the dog (*Canis lupus familiaris*). All animals were raised and kept in intraspecific packs under identical conditions—in accordance with the social structure of the species—in order to control for socialisation with human beings and familiarity with training. We also collected saliva samples of trainers to measure GC and testosterone concentrations, to control for the effects of trainers’ stress levels on the responses of the animals. During the training sessions, separated from pack members, the animals stayed voluntarily close to the trainers and mostly adequately performed requested behaviours, indicating concentration to the task. Similarly to dogs, the salivary cortisol level of wolves–used as an index of stress—dropped during these sessions, pointing to a similar stress-reducing effect of the training interaction in both subspecies. The responses to the requested behaviours and the reduction in salivary cortisol level of wolves and dogs varied across trainers, which indicates that the relaxing effect of training has a social component. This points to another factor affecting the welfare of animals during the sessions, beside the rewarding effect of getting food and control over the situation by successfully completing a task. As all responses performed by the animals corresponded to cues already familiar to them, the reported effects were likely due to the above cited factors rather than to a learning process. Our results support previous findings suggesting that training is a potentially powerful tool for improving welfare in some wild social canids by creating structured and positive interactions between these animals and their human caretakers.

## Introduction

Animals in captivity live in environments that may substantially differ from their natural habitat, in which they developed their behavioural repertoire [1]. In the wild, animals exert control over a significant proportion of their actions. For example, they decide when and how to get involved in particular activities. In contrast, captive animals face a schedule that is usually rigid and temporally regular, giving them few opportunities, for example, to find food and mates, and to exert control over situations to which they are exposed. This situation of reduced choice and controllability may create a context of under- or over-challenge, associated with frustration [2] and chronic stress [3], thereby affecting animal welfare.

The welfare of an animal may be evaluated via the efforts it makes to adapt to its environment [4–5]. Through physiological and behavioural coping strategies, individuals try to gain control over their interactions with the environment to maintain physical and mental homeostasis [6]. Environmental stimuli that lead to an imbalance of homeostasis are usually defined as “stressors” [7]. Behavioural responses to stressors include aggression, increased activity, and avoidance behaviours [6], while physiological response involves increased release of glucocorticoids (GC, e.g. cortisol [7–9]), and increases in heart and breathing rates [10]. Environmental stressors which surpass the coping capacity of the animals–for example, extreme temperatures, or social isolation in a social species—if prolonged or repeated [7] may result in chronic stress and, usually, in poor welfare [10].

The release of GC, which are the metabolic and stress coping hormones, occurs due to a modulation of the stress response system, regulated by the hypothalamic-pituitary-adrenal (HPA) axis [7–8]. Through its physiological and behavioural effects, the release of GC enables animals to cope with threatening or demanding situations [8–9]. However, frequently or chronically elevated GC may have detrimental consequences for learning and memory processes [11], result in the suppression of reproductive and immune systems [9, 12] and cause ill health [7].

The activity of the HPA axis can be non-invasively assessed from saliva samples [13]. Both biogenic amines and GC are released into the blood stream in the short-term–i.e., within seconds to minutes after a stressful event; GC have been preferable to study the effects of stress, among other factors, due to their longer permanence in the blood stream–from minutes up to days [12]. Salivary GC levels accurately reflect the biologically-active portion of total plasma GC, making them suitable for monitoring short-term physiological stress responses [14]. In dogs (*Canis lupus familiaris*), the estimated time for a change in adrenal activity be measured in the saliva is after 15–20 minutes of exposition to stressors, among which sometimes even handling [15, 16]. Regarding wolves (*Canis lupus*), there was so far no data in this estimated time. However, because they are closely related to dogs, we expect a very similar modulation of their HPA axis. A comprehensive understanding of the impact of stressors on animal welfare may be achieved by assessing behavioural (e.g., behaviours indicative of stress) and physiological (e.g., variations in cortisol concentrations, heart or breath rates) stress parameters in tandem.

### Welfare and human-animal interactions

Intraspecific affiliative interactions have long been shown to produce calming effects on captive social animals [17, 18, 19, 20], having their corresponding neural circuitry already unveiled: a number of receptors in brain circuits related to reward or reinforcement were found linked to the formation of bonds in several species [21]. Even non-social species, such as tigers (*Panthera tigris* [22]), and less social ones, such as orangutans *(Pongo pygmaeus* [23]), have been shown to benefit from intraspecific social stimulation in captive settings. Positive human-animal interactions, understood as mutual and dynamic interactions between human beings and animals that have a relaxing effect on animals [24], have been recommended to improve the welfare of captive animals, domesticated or wild. It has been shown that interactions with human beings, in some situations, have the potential to reduce abnormal/stereotypic behavioural patterns and increase the duration of affiliative behaviours with group members [25–28], reduce GC concentrations [29–30], and support the expression of species-typical behaviours [31]. However, other studies have described potential negative effects of intense contact with human beings on the welfare of some mammals [25, 32–38]. Even the mere presence of a human being can be aversive for laboratory primates [39], and farm mammals in petting zoos, albeit domesticated, may show avoidance of contact with visitors [40].

Wild animals, which were not submitted to artificial selection for interacting with human beings, may be overwhelmed by close or frequent contact with them [41–42]. But wild mammals in captivity have also shown either improved welfare by enrichment through human contact [43–44], or increased stress responses [25, 33, 44, 45]. Hogan et al. [41], for example, conducted petting sessions with captive wombats (*Lasiorhinus latifrons*) that reacted to the sessions with avoidance behaviours and an increase in GC concentrations. Although the aversive responses of the animals have decreased as the sessions were repeated, their GC secretion remained high, suggesting that the quiet behaviour of wombats during petting sessions more likely reflected freezing consecutive to elevated stress than relaxation.

Whether interactions with human beings have a positive or a negative effect on animals likely depends on various factors. The level of sociality of the species is possibly one of the factors affecting the results of human-animal interactions, since most studies showing relaxing effects of such interactions have been developed with social species [24]. However, there are also studies reporting neutral or detrimental effects of such interactions on social species [25, 33, 40, 42]. Familiarity with the human partner may also support the relaxing effects of the interactions [46]. In addition, the frequency (i.e., how often they interact), the form and the quality of the interactions (i.e., how voluntary and rewarding the interaction is) an animal has with its caregivers, trainers or researchers also seem to matter [47–48]. However, research investigating the effect of these variables remains scarce, and mainly focused on farm animals [46, 49–51].

Previous work on interactions between human beings and captive wild mammals points at the relevance of the characteristics and experiences of the animals with human beings, for example, how often they have interacted with human beings, how rewarding these interactions were, if the animals were raised by their parents or by people, etc. These factors may contribute to differences in the temperament of the animals and, as a consequence, in their reactions to interactions with human beings [1, 47]. Early socialisation seems to play a crucial role, as demonstrated by a large-scale study with 219 individuals of four zoo-kept species in 46 zoos [47]. For example, wild-born black rhinos (*Diceros bicornis michaeli* and *minor*) and parent-reared maned wolves (*Chrysocyon brachyurus*) showed significantly less affinity to their keepers than their captive-born or hand-reared counterparts [47]. The same study reported a positive correlation between the experience of keepers and the fear of people exhibited by the animals, which these keepers were in charge of, which indicates that certain keeping styles or habits of experienced keepers might be fear-inducing. In order to avoid such detrimental effects on experimental animals, these are frequently hand-reared. In fact, hand-rearing is regarded as essential in some behavioural studies [52, 53, 54], when contact with human beings during testing is inevitable. Although the effects of hand-rearing have been evaluated only with social animals, such procedures are seemingly important for all wild species, as appropriate early socialisation with humans will reduce fearfulness, may promote cooperation from the part of the animals, and ensure safety for human partners [54, 47]. In summary, available data suggest that interactions with human beings can be beneficial, neutral or detrimental for captive wild mammals, depending on context, characteristics of the considered species, and specifics of the interactions (related to the personality of the human partner and the type of relationship developed with the partner).

A type of interspecific interaction that is generally suggested to promote improvements in welfare is Positive Reinforcement Training (PRT), a form of operant conditioning [55–57]. Positive Reinforcement Training is based on reinforcing specific behaviours by rewarding the individuals exhibiting this behaviour. Whereby, an intense and regular contact between trainers and animals may be established during interactions that are strongly controlled by the animal. This technique is being increasingly used as part of protocols for socialising wild mammals for the aims of comparative studies [58]. Although PRT has been shown efficient to reduce stress in some mammals (e.g., chimpanzees (*Pan troglodytes* [59]), gorillas (*Gorilla gorilla* [26]), baboons (*Papio hamadryas* [30]), dogs [29, 60], African wild dogs (*Lycaon pictus* [61]), other studies have reported limited or negative effects of such procedures (e.g., rhesus macaques—*Macaca mulata* [32, 42] and chimpanzees [33]). For most tested species there is a lack of evidence of the efficacy of PRT in improving welfare (e.g. [32, 62], and see [55] for a review). Possibly due to the considerable difficulties of standardising procedures and measuring the multifaceted welfare effects of PRT, studies on its effects in many species have not focused primarily on the effects of the procedure *per se*, but on indirect effects, i.e., on improvements in the specific trained behaviours. In turn, these improvements can promote a reduction in stress level through husbandry facilitation, avoiding the need for aversive procedures [27, 55, 63–69]. Further difficulty faced by these studies is the de-coupling of the effects of the learning process from the effects of the training interactions *per se* [55].

The study of the effects of training on wolves is important for at least two reasons. First, wolves are rather popular and accordingly, many more than the estimated 200000 wolves still living in the wild are kept in zoos or even privately [70–71, 72], despite their shy nature. Although wolves are highly social and cooperative with their pack mates [73, 74], as other untamed/non-socialised wild animals, they seem not well suited for captive life close to human beings [52–75]. During domestication, dogs have diverged from wolves in many ways, including in their behaviour and ecology [76, 77]. For example, even the social organisation of wolf packs and feral domestic dogs differ: wolves are more cooperative in hunting, offspring raising, and in defending a territory [78]. Second, wolves have been trained and used in captive settings as experimental subjects in comparative studies, primarily in comparison to dogs, in order to study the evolution of social behaviour and cognition [79–82]. Currently it is unknown whether intensive socialisation with human beings–through the reduction in fear and increase in cooperation this procedure is claimed to promote [53]—may allow wolves to benefit from the relaxing and stress-reducing effects of training in a way similar to that of human-socialised dogs. Alternatively, wolves, known for their shyness, might not benefit from training, and show an increase in GC concentrations and a higher frequency of behaviours non-related to training–i.e., behaviours different from the ones being requested by the trainers.

In this study we aimed at investigating the behavioural (behaviours indicative of cooperation or stress) and physiological (variations in cortisol concentrations) effects of PRT on human-socialised wolves, a wild species. Uniquely, we compared the effects of PRT on wolves to the effects on dogs raised and kept under identical conditions. As the positive effects of PRT have been repeatedly demonstrated in dogs—with no reported sex- or age-related differences [83–85], comparing the responses of dogs and wolves to the same procedures would give us an idea of the magnitude of this effect on wolves. Beyond the implication for welfare, a stress-reducing effect of training on wolves would support the validity of the current practice of studying the evolution of cognition by comparing the social behaviour of equally raised and kept wolves and dogs in interactions with human beings.

## Materials and Methods

### Ethics Statement

All study animals were kept at the Wolf Science Center (www.wolfscience.at), located in Game Park Ernstbrunn in Austria (License No.: AT00012014). The CITES (www.cites.org) import permits for the animals used in this study are: 2008: Zoo Herberstein, Austria: AT08-B-0998, AT08-B-0996, AT08-B-0997; 2009: Triple D Farm, USA: AT09-E-0018; 2012: Minnesota Wildlife Connection, USA: 12AT330200INEGCJ93. The animals were housed in accordance with the Austrian Federal Act on the Protection of Animals (Animal Protection Act—TSchG, BGBl. I Nr. 118/2004). All animals tested in this study could voluntarily choose to enter the experimental enclosure and to participate in a training session. If the animals entered the experimental enclosure but were not motivated enough to participate–i.e., if they failed to respond to the cues during the first minute of the session—they were shifted back into their home enclosure. The methods used in this study were strictly non-invasive. Hence, in accordance with the Austrian Animal Experiments Act (BGBl. I Nr. 114/2012, Tierversuchsgesetz 2012 –TVG 2012) no ethical approval was required, but we obtained one from the University of São Paulo, Brazil (Committee of Ethics for Animal Research from the Institute of Psychology, University of São Paulo, Brazil, approval number 016.2009).

**Fig 1:**
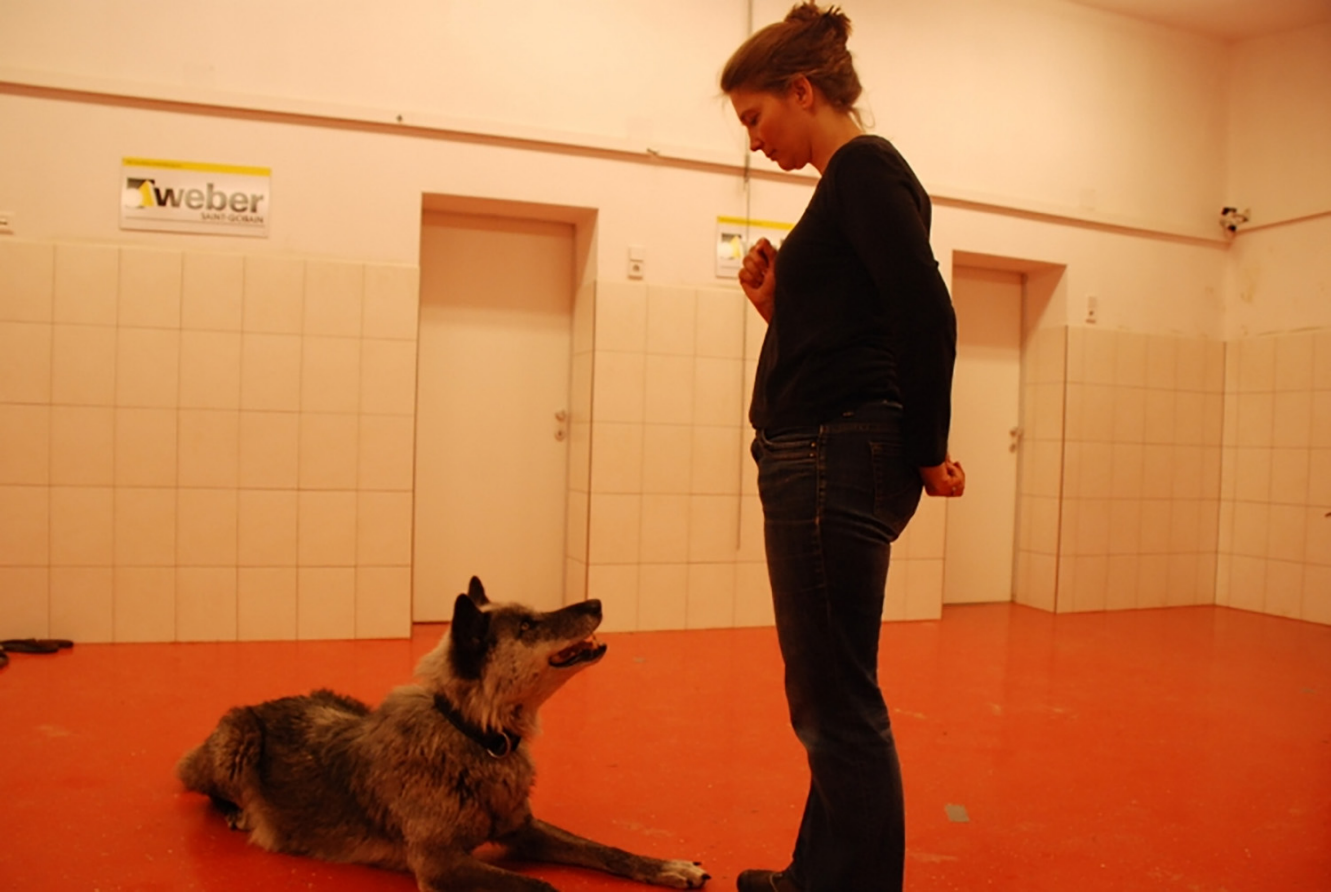
Picture of a training session showing the female wolf Shima performing the response to the cue *down*, within one metre from the trainer FR.

The individual pictured in Fig 1 (FR) has given written informed consent (as outlined in PLOS consent form) to publish her picture.

### Subjects

We studied nine timber wolves and nine mixed breed dogs. The characteristics of the animals, such as sex and age are reported in Table 1. All the animals were captive-born and hand-reared with the participation of all trainers contributing to this project. This procedure allowed us to control for developmental experiences, human socialisation and familiarity with training. Hand-raised wolves and dogs had daily contact with human beings, 20 to 24 hours a day, 7 days a week, from their first week of age until the age of 16 to 20 weeks, which also included bottle feeding and, later, hand feeding. After this period of time, the animals were integrated into a pack with older conspecifics, and interactions with human beings were reduced to four to five days per week, approximately 30 min per day. From their third week of life, all animals consistently received PRT, becoming familiarised to cues such as “sit”, “down”, “stay”, “roll-over”. In all training sessions the responses of the animals were rewarded through a continuous reinforcement schedule, and behaviours were shaped with a secondary reinforcer (clicker). Besides that, each animal participated in several weekly food-rewarded behavioural tests. Each animal took voluntarily part in all training sessions; the animals were called by name and came to the door of the training room. No interactions of the animals with human beings involved domination, punishment, or any other negative interventions, and when items had to be taken away from the animals, they were generally exchanged for food.

**Table 1:** Characteristics of the studied animals.

Subspecies	Name	Sex	Age at first testing (months)
Wolf	Aragorn	Male	24
Wolf	Shima	Female	24
Wolf	Kaspar	Male	24
Wolf	Tatonga	Female	11
Wolf	Nanuk	Male	11
Wolf	Geronimo	Male	11
Wolf	Yukon	Female	11
Wolf	Cherokee	Male	11
Wolf	Apache	Male	11
Dog	Meru	Male	43
Dog	Nuru	Male	26
Dog	Zuri	Female	26
Dog	Nia	Female	25
Dog	Layla	Female	24
Dog	Rafiki	Male	12
Dog	Alika	Female	12
Dog	Maisha	Male	11
Dog	Kilio	Male	11

Both wolves and dogs lived in packs with conspecifics, in enclosures of 3500 and 900 m^2^ respectively, at the Wolf Science Center, Ernstbrunn, Austria. The animals had a diet of meat, milk products and dry food throughout the study period. As the two studied subspecies have different feeding requirements, each subspecies was fed following its own feeding rhythm: wolves had major meals of carcasses once to three times per week and dogs received smaller portions of dry food or meat every day. Water was permanently available for all animals.

### Procedures

#### Saliva collection

In the training sessions, we used samples of saliva of the animals for a physiological assessment of stress, through the measurement of cortisol concentration. Previously to the training experiment, all animals were trained for saliva sampling with the use of PRT. Saliva collection was performed by the introduction of a surgical hydrocellulose sponge (Sorbette, by Salivette ®) into the cheek pouch of the animal for as many times as necessary to collect enough saliva (two soaked sponges). During saliva collection, as well as during the training sessions, the animals were rewarded only with cheese, to control for the influence of proteins in the saliva samples [86]. Saliva collection was performed a few (2 to 10) minutes before and 15 minutes after the end of each training session. Immediately after saliva collection, the sponges were transferred into a plastic tube (Sarstedt ®) and stored at –20°C until analysis. As there was, so far, no estimation on the time of delay between exposition to stressors and detectable changes in salivary cortisol concentrations in wolves, we performed a validation procedure, which results pointed to the same time lag as the one recorded in dogs (i.e., 15–20 minutes, [S1 Text]). The procedure of attracting one animal and collecting its saliva took less than 10 minutes, around 4 minutes in most sessions, and thus the adrenal activity measured in the first sample could reliably be considered as not related to the handling procedure.

Saliva samples of the trainers were also collected before each training session, using the same material as the one used for the collection of saliva from dogs and wolves. The saliva samples of trainers were analysed to measure GC and testosterone concentrations, to control for the effect of the stress level of the trainer on the response of the animals [87].

#### Training sessions

Each studied animal took part in 15 different training sessions of five minutes each, for a total of 270 sessions. The training sessions were videotaped, and took place between May 2010 and March 2011. Each trainer (BB, FR, KK, RT, ZV) was equally involved in the training of every one of the animals, conducting three sessions with each studied animal. The sessions with each animal were run on average within four months. Each animal participated in the sessions in isolation from the pack, between 8:30 h and 17:00 h, in a training room (63.6 m^2^) located next to their home enclosure. The training room was empty, except for an elevated platform on one side, and did not allow visual contact with other animals or human beings. No animal was trained more than once a day. Each trainer, however, worked with four to nine animals on each training day, with the order of animals randomised across sessions.

Before training an animal, the trainer collected his/her own saliva, called the animal, led it into the training room and collected its first saliva sample. As soon as the saliva sample of the animals had been taken and stored, the trainer started giving cues (PRT, Fig 1) continuously, in a freely chosen order for five minutes. Although there was no pre-defined order for the cues in the sessions, the trainers used mostly the same proportions of each cue. The behaviours requested were sitting, laying down, turning around, walking around the trainer, giving the paw, allowing the placement of a muzzle, allowing the placement of a harness, rolling, standing, staying and looking into the trainer’s eyes. Using a continuous reinforcement schedule the animal was rewarded with pieces of cheese for presenting a correct response. If the animal failed to perform the requested response, the cue was given again after five seconds.

After the end of the session, the trainer and the animal interacted freely to keep the subject entertained for 15 more minutes until the second saliva sample was taken from the animal. Finally, the animal was sent back to the pack.

### Data Analyses

All evaluated behavioural parameters–behaviour and orientation of the animals, requested cues and distance between the animal and the trainer—were coded through continuous sampling of the videos, using the program Solomon Coder (Beta version 11.03.28, 2006–2011 by András Péter).

Besides the responses to the cues, non-training behaviours (NTB) exhibited by the animals were also recorded. Non-training behaviours were all behaviours that did not contribute to the training process and took the animal, or its attention, away from the trainer. These behaviours were considered as indicators of lack of attention, boredom or of an increase in stress levels [88, 89], indicating that the animal was not focused on the activity.

The observed NTB were: 1—jumping on the trainer: the animal is either standing on its hind legs or jumping, with its four legs leaving the ground, and touches the trainer with its forelegs; 2 –leaving: the animal moves away from the trainer, abandoning the interaction, and 3 –exploring: the animal sniffs the ground or the walls.

Each video was watched three times. During the first viewing, the behaviour and orientation of the animal were registered. The second viewing aimed at coding the trainer’s cues. During the third viewing, the distance between the members of the dyad was recorded. The distance between the animal and the trainer was classified into three categories: a) less than one metre, b) between one and three metres, c) more than three metres.

In terms of behaviour, the following response variables were measured: a) proportion of time the animal spent oriented towards the trainer (the orientation of its head deviating less than 10° from the trainer’s face) [90]; b) proportion of time the animal spent within 1m of the trainer; c) mean latency to respond to the cue at first request; d) proportion of cues correctly responded at first request; e) number of cue repetition–if one cue had to be repeated because the animal did not respond to it at first request, only the responded cue was considered as correct; all cues that were not responded were coded as “not executed”; f) proportion of time spent exhibiting NTB.

As a physiological response variable, the variation between the salivary cortisol (SC) concentration of the animals before and after the sessions (measured as nanograms per ml of saliva–ng/ml) was used. The absolute values of SC of wolves and dogs, characteristic of each subspecies, were not compared; all comparisons were performed between the mean SC concentrations in the samples taken before and after the sessions. From the saliva samples of the trainers, we measured testosterone (picograms per ml of saliva–pg/ml) and SC (nanograms per ml of saliva–ng/ml) concentrations. All saliva samples were analysed using an enzyme immunoassay as described by Haubenhofer and Kirchengast [35], previously validated by Patzl [91].

As each animal was trained three times by each trainer (replication), a Linear Mixed Model was used [92], in which the animals themselves and the interactions between the trainer and the animal were considered as random effects, using the following covariates as fixed effects: 1) fasting, i.e., the number of days since the last full meal; 2) age of the animal (in months); 3) sex of the animal; 4) subspecies; 5) session period (morning or afternoon); 6) weather conditions–i.e., if the weather was (a) sunny, (b) cloudy, (c) rainy or (d) snowy; 7) SC concentration of trainer, and 8) testosterone concentration of trainer. The influence of the variable “fasting” was investigated only in wolves, as dogs were fed on a daily basis.

Behavioural and hormonal results are reported as mean ± standard error. Statistical analyses were performed with the software SPSS version 20. All videos were coded by the same observer (ASV), who was familiar with the animals and the coded behaviours. To confirm scoring consistency, 20% of videos were also coded by another observer (DS), and the records were correlated [93, 94]; Spearman’s rank correlations (r_S_) per video were all above 0.85.

## Results

### Variables related to stress response

**Fig 2:**
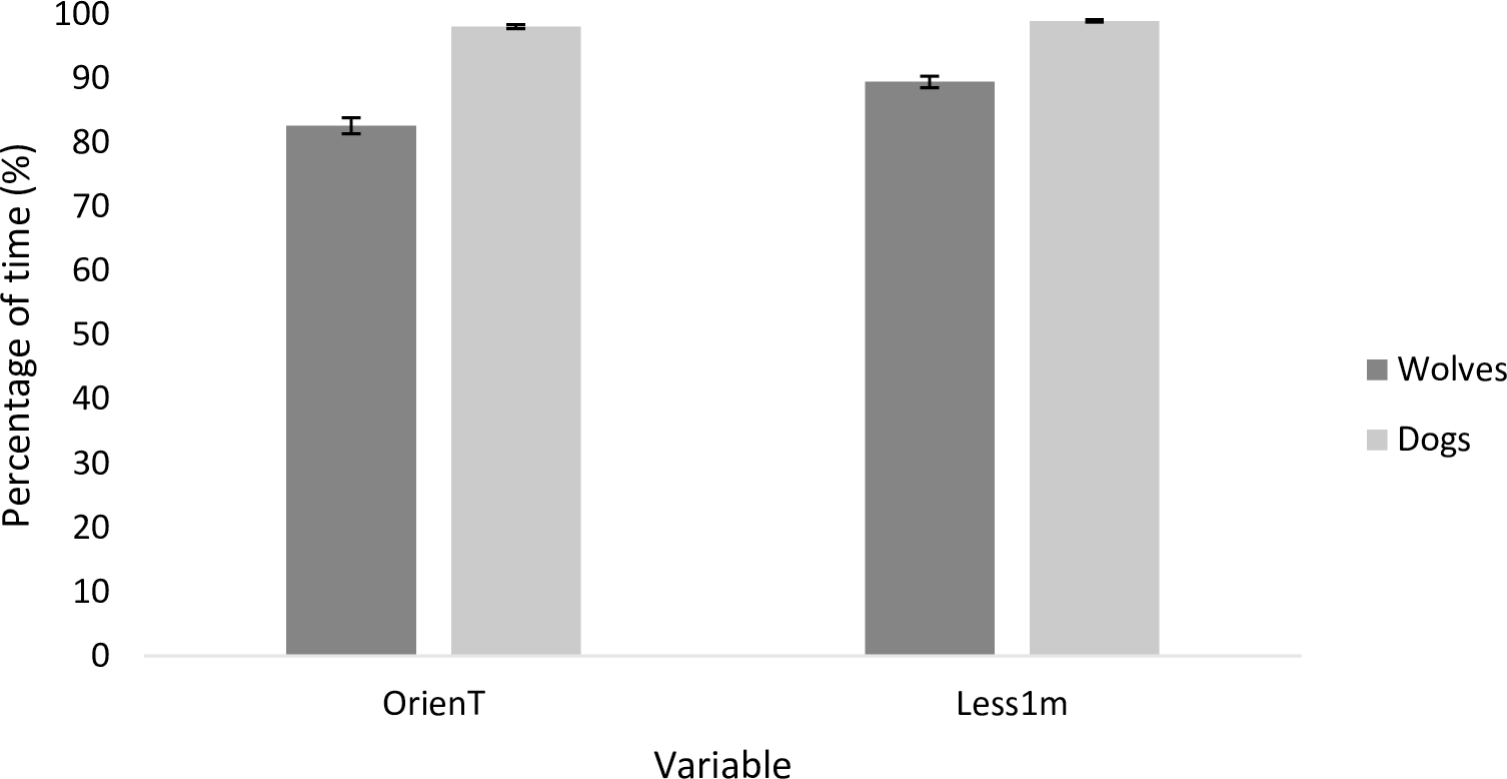
Mean percentage of time (±SE) spent by dogs and wolves within one metre of the trainer (Less1m) and with their face oriented towards the trainer (OrienT) during the training sessions.

**Fig 3:**
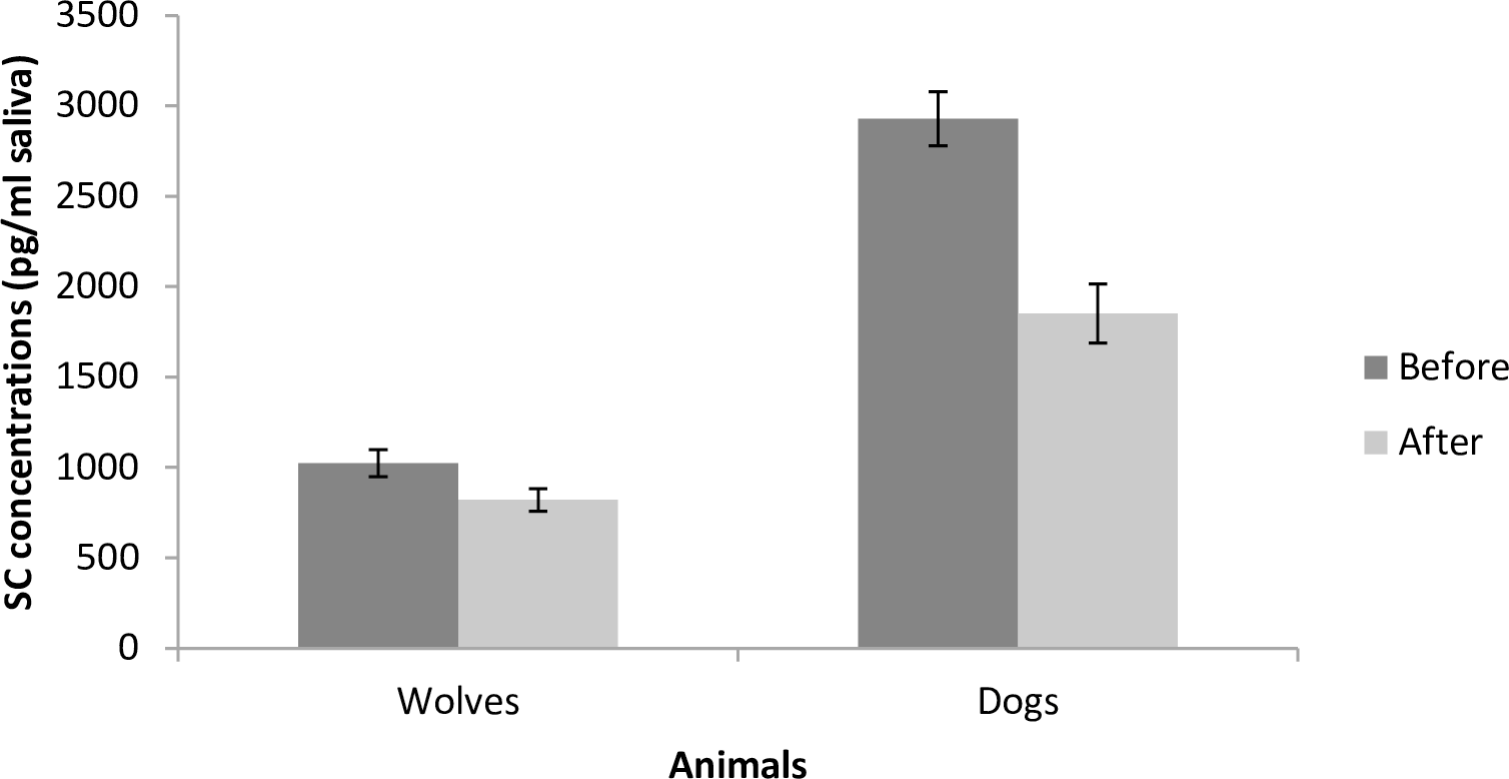
Mean SC concentrations (±SE) in samples collected from wolves and dogs (1) before and (2) 15 minutes after the end of the training sessions.

The animals showed no behaviour suggesting distress (i.e. panting, lip licking, pacing, tail tucked between legs etc. [88, 89]) during the sessions. Both wolves and dogs spent most of the time of the session voluntarily within one metre of the trainer and were attentive to him/her. Dogs spent significantly more of the training time within one metre of the trainer than wolves (99±0.2% versus 89.5±0.9%; *t* = 4.760, *p* < 0.0001, Fig 2). There was a tendency of decrease in the time the animals spent within one metre of the trainer as the trainer’s SC concentrations increased (*t* = 1.924, *p* < 0.056). In dogs and wolves together, 16% of variability in the time spent close to the trainer was explained by the identity of the trainer. No other measured variable was affected by the measured SC concentrations of the trainers. Dogs spent significantly more time with their face oriented towards the trainers than wolves (98.1±0.3% versus 82.7±1.2%; *t* = -7.870, *p* < 0.001, Fig 2), and 11% of orientation variability for dogs and wolves together was due to trainer identity. The longer the period the wolves had been fasting before the training session, the greater the proportion of time they spent oriented towards the trainer (*t* = 2.155, *p* < 0.033). A proportion of the variability in the effect of fasting on the wolves’ orientation (5.9%) was affected by trainer identity. Behaviours not related to training were infrequently observed (<10% of the time; i.e., on average less than 30 seconds per session). Wolves showed explorative behaviour towards the test room for a greater proportion of the session than dogs (7.2±0.6% versus 0.8±0.1%; *t* = 6.221, *p* < 0.001). Also, wolves spent more time temporarily quitting the training situation than dogs (1.6±0.2% versus 0.03±0.02%, *t* = 6.095, *p* < 0.001). Regarding jumping, there was no difference between wolves and dogs (wolves 0.4±0.06%, dogs 0.2±0.004%, *t* = 0.977, *p* = 0.341). After each training session, the SC concentration of animals was reduced compared to the measurement taken before the training session in both wolves and dogs (*t* = -2.864, *p* = 0.004, Fig 3; wolves before training–BT = 1023.03 ± 75.99 ng/ml; wolves after training–AT = 820.13 ± 64.03 ng/ml, a decrease of 19.83%; dogs BT = 2280.87 ± 153.2 ng/ml; dogs AT = 1851.99 ± 162.9 ng/ml, a decrease of 18.8%). The drop in GC concentrations was observed in most wolves (eight out of nine) and dogs (seven out of nine).

### Variables related to the performance of the animals during the training sessions

**Fig 4:**
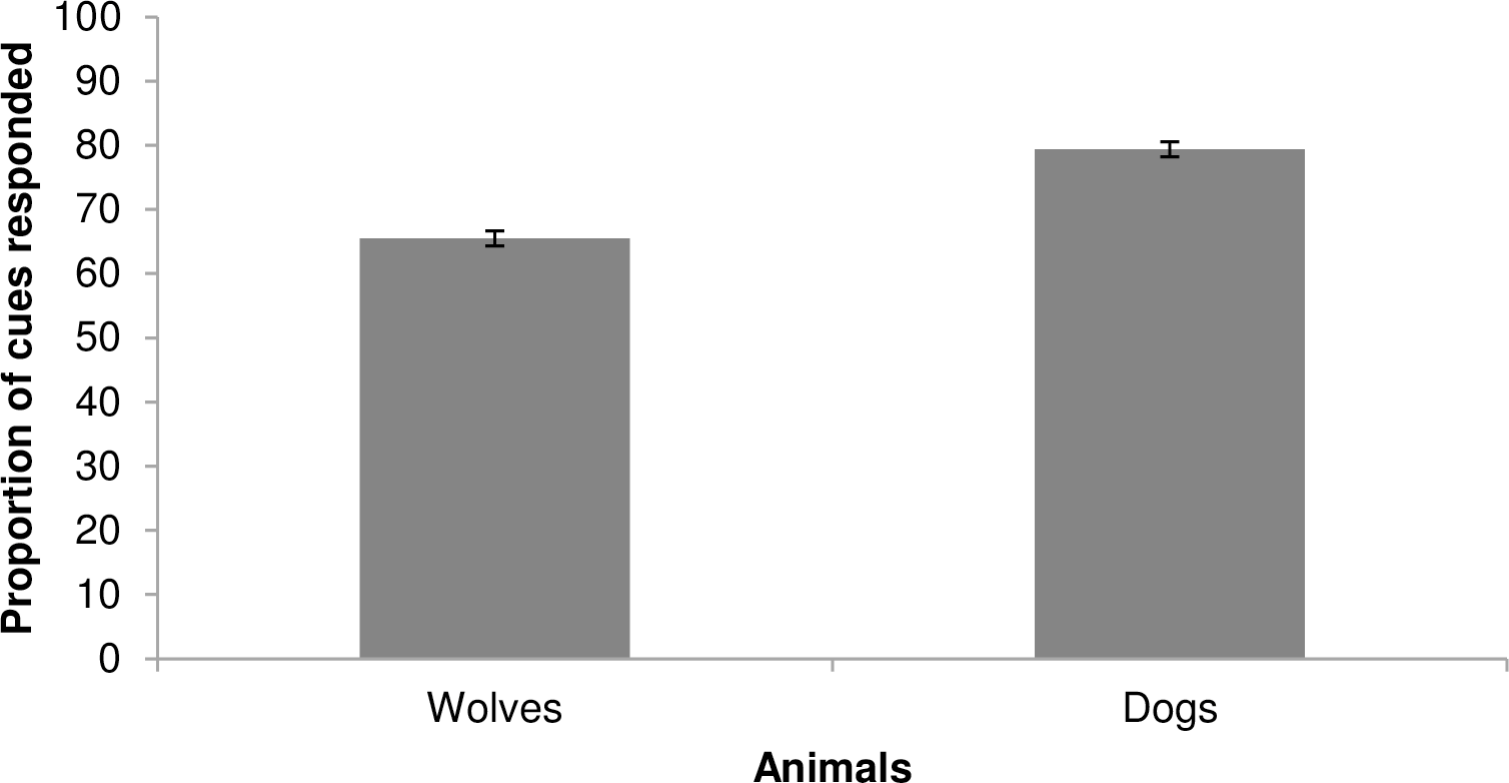
Mean proportion (±SE) of cues responded by dogs and wolves during the training sessions.

**Fig 5:**
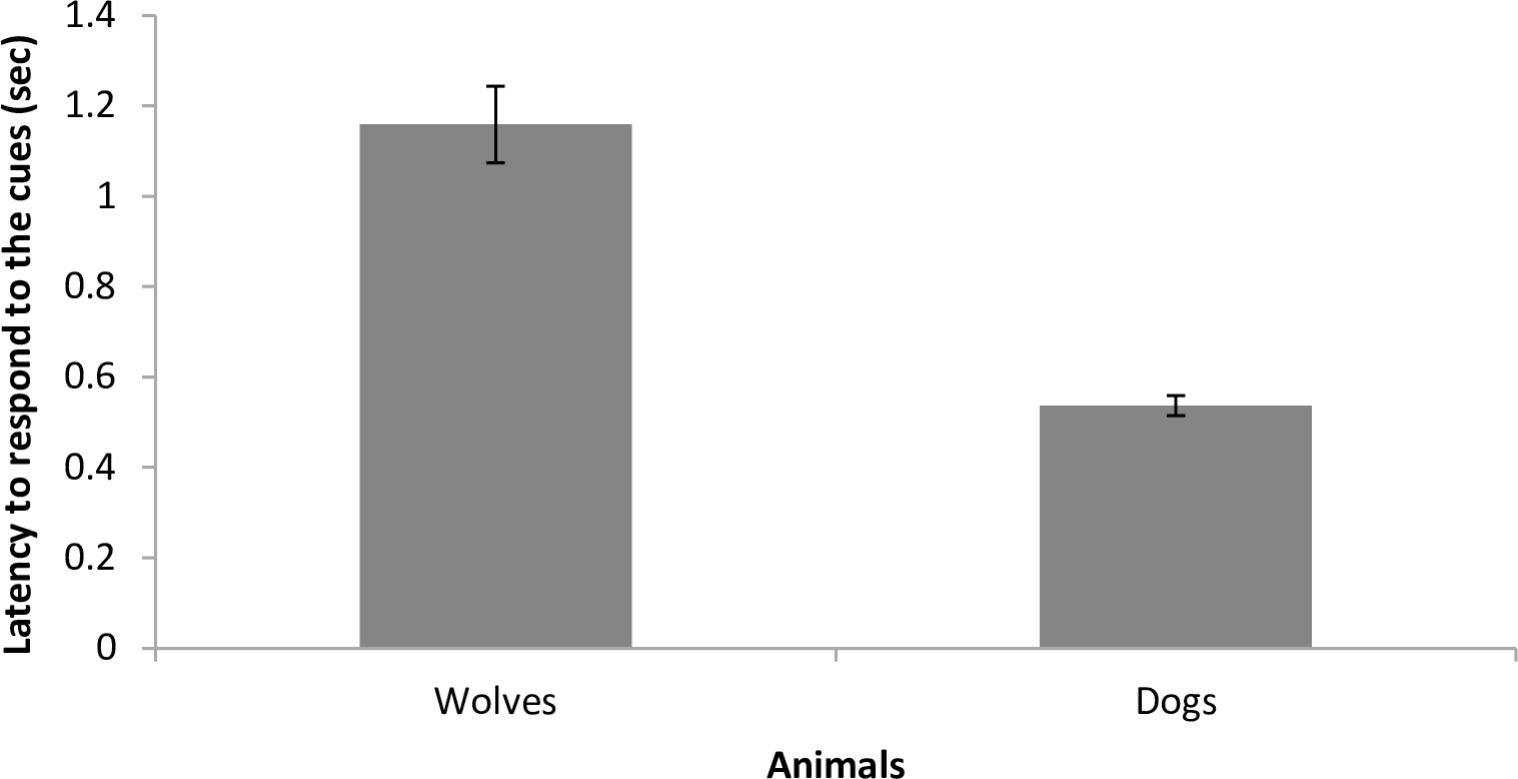
Mean latencies (±SE) of dogs and wolves to respond to the cues during the training sessions.

Dogs responded to a greater proportion of cues than wolves (79.4±1.2% versus 65.5±1.2%; *t* = -5.499, *p* < 0.001, Fig 4). Trainer identity explained 22.8% of the variability in performance of dogs and wolves. In addition, although both dogs and wolves responded to the cues promptly (mean latency 0.5±0.02 seconds in dogs and 1.2±0.1 seconds in wolves), dogs were significantly faster than wolves (*t* = 5.502, *p* < 0.001, Fig 5). The older the animal, the shorter the latency to respond to the cues (*t* = -3.510, *p* = 0.003) and the fewer repetitions were necessary to get a response, in both wolves and dogs (1.5±0.04 times, *t* = -2.878, *p* < 0.009). In dogs and wolves together, trainer identity explained 16.6% of the variability in latency for response to the cue and 16.4% of variability in cue repetition. The mixed models are available on S1 Table.

## Discussion

Both wolves and dogs intensely attended the training in terms of proximity, orientation towards trainer and responses to the cues. In addition, we recorded a reduction in SC concentrations in both dogs and wolves, in response to the training sessions. These results, in connection with the low rates of NTB observed, suggest that the animals were motivated to participate in the training interactions and that these procedures may have positively affected their wellbeing. Dogs and wolves performed very similarly. However, dogs were slightly, but still significantly more attentive and responsive, and faster than wolves.

Training has been claimed to be an animal welfare promoter in some species since it affords learning, defined as a change in behaviour resulting from practice or experience [55, 84], as shown in chimpanzees [59], gorillas [26], baboons [30], dogs [29, 60] and African wild dogs [61]. This effect is connected to the understanding of the relationship between an action and a reward, which elicits a positive affective state [84]. In our study, however, all behaviours requested during the training sessions were already familiar to the animals. Once an animal has understood the connection between a cue and the correct behavioural answer to the cue, this is no longer a learning process. Therefore, our data allowed us to de-couple the effects of learning from the effects of the training interaction. Our results suggest that the measured decrease in SC levels was not due to a learning process but rather to mastering a task in a social setting together with a trainer, with whom the animals were well socialised.

Once an individual is rewarded upon the correct execution of certain behaviour on cue, it exerts control over its environment [95–96]. Control is claimed as psychologically and physiologically important [97] since the immediate psychological goal of behaviour is to exert control over a situation to which an individual is exposed (e.g. [98–99]). In the wild, animals are able to control the amount and quality of stimulations they receive, by performing various behaviours such as approaching, attacking or hiding from situations, until the stimulation they receive is at an acceptable level, or until their expectations of the stimulation are met. The matching of behaviours performed with their expected consequences are achievements known to be emotionally positive [100], a process possibly linked to the activation of the brain reward systems [84, 101–102]. Besides, the age effect on the quality of performances observed in our study parallels other studies on cooperation and a body of experience of people working with domestic dogs; generally, attentiveness and impulse control in dogs seem to improve particularly over the first two years of life [80, 103].

Beyond control over the environment, it appears that the relationship between the animals and their trainers and/or the manner in which the interaction is conducted—i.e. the tone of the trainers’ voice, the rhythm of the session and other aspects related to the personality of the trainer—also played a significant role in the relaxing effect of the interaction. Importantly, the effect of the identity of the trainer was not due to his/her stress level (measured by his/her SC concentration) at the time of the session. Being comfortable in the situation and having a good relationship with the trainers likely contributed to the fact that the animals participated in our training sessions voluntarily and intensely, and that their SC levels dropped during the sessions, in agreement with previous results [26, 30, 46, 59, 104].

Our results suggest that controllable interactions in a cooperative setting with human partners—with whom the animals are closely socialised—may increase the welfare of wolves and dogs, in accordance with previous findings [46, 56, 88, 105–107]. In captive primates, desirable changes in the behaviour–i.e., reduction in the frequency of abnormal behaviour, and in physiological responses–levels of GC concentrations—have been found when the animals could exert control over aspects of their environment such as food delivery [108] or highly disturbing noise [109]. African wild dogs showed a reduction in the frequency of pacing in response to PRT, possibly because the requested behaviours replaced highly motivated species-typical behaviours–prevented by the captive environment, such as hunting—and, consequently, matched a behavioural need for expressing such behaviours [61].

An alternative explanation of the observed stress-reducing effects of training is the separation of the animals from the pack for the duration of the training sessions. In animal groups, during conflicts, individuals interact with each other aggressively, generating social stress. In these situations, even dominant animals may show signs of distress [110]. Aggressive interactions tend to be more frequent in captivity, where animals have limited possibilities of conflict reduction through spacing out [58, 111]. Considering the constraints of captivity, because the individuals would not be exposed to social tension for the time of the session, they might have experienced adrenal deactivation. However, this is unlikely, because the animals showed no reluctance to return to their packs after training.

Also, it is possible that the interest of the animals in the training sessions was strongly motivated by the food rewards provided during these sessions [112]. This hypothesis is supported by our results, as the proportion of time wolves spent oriented towards the trainer increased with the fasting period. However, previous work on African wild dogs refutes this hypothesis, as the time spent exhibiting pacing behaviour was significantly reduced after training sessions as compared to control sessions, in which the canids were fed without training [61]. Obtaining the food reward is certainly a factor for responding, but we suggest that control and social factors may play a role as well. This is supported by the fact that although all trainers have worked with food rewards, still a great variability (ranging from 5.3 to 22.8%) in virtually all tested parameters was caused by trainer identity-. As reported in other studies [113–114], attentiveness toward potential partners seems to vary according to the relationship the subject has with its partner. In other words, attentiveness is affected by a social interaction factor.

Our findings regarding the role of individualised animal-trainer relationships confirm that appropriate training procedures may be seen as positive, cooperative human-animal interactions [46]. Several studies on farm animals have shown that stockpersons differ in the way they interact with their animals, and this difference has an effect on the value of the relationship (negative, neutral or positive [46, 51]) between them and their animals. It is known that captive wild mammals also generally distinguish between different keepers and respond to them in a specific way [47–48]. Some evidence even indicates that the welfare of captive mammals scales with the time caretakers spend with them (chimpanzees [45]; callitrichids—*Callithrix jacchus* [104]; small felid species—*Felis spp* [115]), particularly if they engage in structured activities such as training (callitrichids [63, 116]).

The high degree of cooperation recorded in the sessions with wolves–i.e., the great proportion of correct responses, low latency to respond to the cues and low frequency of NTB—suggest a willingness of these animals for social interactions, even with partners from another species. These results corroborate other studies suggesting that the promptitude of dogs to cooperate with human beings might have evolved on the basis of wolf-wolf cooperation [103, 117–118]. That we found no fundamental difference between the readiness of dogs and wolves to comply with human instructions suggests domestication did not crucially contribute to the origins of the abilities of dogs to interact with human beings. Our findings show that regularly trained wolves that are intensively socialised with human beings can benefit from the relaxing effects of training to the same degree as dogs, and indicate that given proper socialisation, the cooperative attitude of wolves is sufficient to ensure response to human cues in exchange for food reward. This would not exclude, however, that domestication did affect responsiveness and attentiveness to human cues in dogs [53, 119], which may be reflected in the minor, but significant observed differences in training performances.

Non-human animals are an integral part of experimental research. The success of experiments with animals, i.e. the comparability and repeatability of results may crucially depend on keeping and handling procedures, and on how the animals are prepared to collaborate in the testing / training sessions–including the important stage of socialisation of the animals with human beings. Stress response may negatively affect the performance of animals in complex cognitive tasks [120], such as spatial memory [121–123]. This is where the quality of scientific results and animal welfare meet. Our results bear implications for animal welfare, pointing at training sessions to provide adequately socialised wild canids with positive interspecific interactions, contributing to reduce stress in captivity. Raising and maintaining animals in good physical and mental conditions, including friendly and relaxing interactions with human beings, have the potential not only to benefit welfare, but also to make the animals adequate partners for comparative studies. Our behavioural and physiological results highlight short-term effects of PRT on the stress levels of dogs and socialised wolves. Future research comparing trained and untrained canids, kept under the same conditions and evaluated for longer periods of time may shed more light in the matter.

## Supporting Information

S1 Table Final reduced models of effects of age, sex, subspecies, session period, weather, fasting (only for wolves), trainer cortisol and testosterone levels on the response variables exploring, jumping, leaving, orientation towards the trainer, Less1m, mean latency, cue repetition, responded cues and cortisol concentration. Non-significant effects of fixed effects and interactions between them are not shown because they were removed in the model selection process.
(DOCX)

S1 Text Validation of the appropriate timing for the measurement of salivary cortisol levels in wolves (DOCX)
