# Supplementary material for: Training Reduces Stress in Human-Socialised Wolves to the Same Degree as in Dogs
Source: PLoS One. 2016 Sep 9;11(9):e0162389. doi: 10.1371/journal.pone.0162389 (PMC5017772; doi:10.1371/journal.pone.0162389)
Supplement: S1 Table — Non-significant effects of fixed effects and interactions between them are not shown because they were removed in the model selection process. (DOCX) [file pone.0162389.s001.docx]

| S1 Table. Final reduced models of effects of age, sex, subspecies, session period, weather, fasting (only for wolves), trainer cortisol and testosterone levels on the response variables exploring, jumping, leaving, orientation towards the trainer, Less1m, mean latency, cue repetition, responded cues and cortisol concentration. Non-significant effects of fixed effects and interactions between them are not shown because they were removed in the model selection process. | | | | | |
| --- | --- | --- | --- | --- | --- |
|  |  |  |  |  |  |
| **Response variables** | **Estimate** | **Std. Error** | **df** | **t** | **p** |
|  |  |  |  |  |  |
| **Exploring** |  |  |  |  |  |
| Intercept | 2.416296 | 2.198918 | 16 | 1.099 | .288 |
| Subspecies^a^ | 19.345185 | 3.109740 | 16 | 6.221 | .000 |
| **Jumping** |  |  |  |  |  |
| Intercept | .876673 | .567706 | 15.462 | 1.544 | .143 |
| **Leaving** |  |  |  |  |  |
| Intercept | .097778 | .532821 | 88 | .184 | .855 |
| Subspecies | 4.592593 | .753523 | 88 | 6.095 | .000 |
| **OrientT** |  |  |  |  |  |
| Intercept | 294.327407 | 4.167379 | 16 | 70.627 | .000 |
| Subspecies | -46.385185 | 5.893563 | 16 | -7.870 | .000 |
| Intercept (wolves) | 225.757388 | 11.785672 | 84.998 | 19.155 | .000 |
| Fasting | 6.038211 | 2.802149 | 122.792 | 2.155 | .033 |
| **Less1m** |  |  |  |  |  |
| Intercept | 300.866432 | 4.559217 | 23.828 | 65.991 | .000 |
| Subspecies | -27.725424 | 5.824296 | 15.995 | -4.760 | .000 |
| Cortrein | -.168926 | .087805 | 229.626 | -1.924 | .056 |
| **Mean latency** |  |  |  |  |  |
| Intercept | .899652 | .120469 | 16.253 | 7.468 | .000 |
| Subspecies | .475373 | .086396 | 14.809 | 5.502 | .000 |
| Age | -.016290 | .004641 | 16.808 | -3.510 | .003 |
| **Cue repetition** |  |  |  |  |  |
| Intercept | 1.874419 | .154849 | 19.538 | 12.105 | .000 |
| Age | -.020004 | .006951 | 20.436 | -2.878 | .009 |
| **Responded cues** |  |  |  |  |  |
| Intercept | 46.288889 | 1.872683 | 16 | 24.718 | .000 |
| Subspecies | -14.562963 | 2.648373 | 16 | -5.499 | .000 |
| **Cortisol** |  |  |  |  |  |
| Intercept | 1656.195001 | 175.562438 | 21.062 | 9.434 | .000 |
| Sample^b^ | -320.474929 | 111.886198 | 410.054 | -2.864 | .004 |

| ^a^Subspecies **-** wolf or dog: wolf is the reference group  **^b^**Sample - before or after: after is the reference group  Exploring: the animal sniffs the ground or the walls;  Jumping: the animal jumps at the trainer;  Leaving: the animal moves away from the training situation, abandoning the interaction. OrienT: proportion of time the animal spent oriented to the trainer  Less1m: proportion of time the animal spent within one metre of the trainer;  Mean latency: mean latency to respond to the cues;  Cue repetition: mean number of cue repetitions;  Responded cues: mean number of responded cues per session;  Cortisol: variation in concentrations of cortisol of the animals between the sample collected before and after the training session. |
| --- |
