# Supplementary material for: Training Reduces Stress in Human-Socialised Wolves to the Same Degree as in Dogs
Source: PLoS One. 2016 Sep 9;11(9):e0162389. doi: 10.1371/journal.pone.0162389 (PMC5017772; doi:10.1371/journal.pone.0162389)
Supplement: S1 Text — (DOCX) [file pone.0162389.s002.docx]

**S1 -** S1 Text. Validation of the appropriate timing for the measurement of salivary cortisol levels in wolves

In dogs, the estimated time for a change in the activity of the adrenal gland to be measured in the saliva is after 15-20 minutes of handling or exposition to stressors [Vincent and Michell,1992; Hennessy et al. 1998]. Regarding wolves, so far there were no data in this estimated time. In order to define the adequate timing for saliva sampling of wolves, we performed a validation experiment.

*Subjects*

For this validation, we studied a subsample of the group of wolves: Shima, Aragorn and Kaspar, at the age of 25 months.

*Procedures*

There were two conditions. In the baseline condition, every animal was conducted individually to a training room and had its saliva collected every 10 minutes, for 40 minutes. Saliva collection was performed through the introduction of a surgical hydrocellulose sponge (Sorbette, by Salivette ®) into the cheek pouch of the animal for as many times as necessary to collect enough saliva (two soaked sponges), a procedure to which the animals had been trained (PRT). During saliva collection, the animals were rewarded only with cheese, in order to control for the influence of proteins in the saliva samples [66]. After the second sampling, the animal was left alone in the room for 1 minute, after which it was reunited with the trainer and sampling continued until the end of the sampling period (40 minutes). One week later (noise condition), another set of samples was collected, following the same procedures. However, in this condition, after the second collection, when the animal was left alone in the room, a loud song (drums) was played for 1 minute.

*Results and discussion*

There were peaks in the SC concentrations of all three animals (increases in SC concentrations= Kaspar - 48.1%, Shima - 46.4% and Aragorn - 45.5%, Fig. 1) after 15-20 minutes of the song playing. These results indicate variations in SC could be reliably measured in saliva of wolves in the samples are collected after the same time lag used with dogs [Vincent and Michell 1992; Hennessy et al. 1998].

*Figure 1 – Concentrations of salivary cortisol of the wolves a) Aragorn, b) Kaspar and c) Shima during the validation experiment, for evaluation of adrenal response during a short-time stressor. Baseline data were collected during the simple isolation of each individual in a room. Noise refer to the same procedure followed by the playing of a loud noise for 1 minute.*

*References*

Hennessy, M.B., Williams, M.T., Miller, D.D., Douglas, C.W., and Voith, V.L. (1998). Influence of male and female petters on plasma cortisol and behaviour: Can human interaction reduce the stress of dogs in a public animal shelter? Applied Animal Behaviour Science *61*, 63-77.

Vincent, I.C., Michell, A.R., 1992. Comparison of cortisol concentrations in saliva and plasma of dogs. Research in Veterinary Science 53, 342–345.
